# Tactics and Strategies Operating in Everyday Life Among Women with Experiences of Forced Migration

**DOI:** 10.1007/s44474-026-00011-5

**Published:** 2026-10-09

**Authors:** Sarah Scheer, Eric Asaba, Thomas Buerk, Manuel Guerrero, Margarita Mondaca

**Affiliations:** 1 NVS, Karolinska Institutet, Stockholm, Sweden; 2 Department of Health and Rehabilitation, Sahlgrenska Academy, University of Gothenburg, Gothenburg, Sweden; 3 NVS, Karolinska Institutet, Stockholm, Sweden; 4 Centre for Research Ethics & Bioethics (CRB), Uppsala University, Uppsala, Sweden; 5 Department of Bioethics and Medical Humanities, Faculty of Medicine, University of Chile, Santiago, Chile; 6 Department of Community Medicine and Rehabilitation, Umeå University, Umeå, Sweden

**Keywords:** Power dynamics, Intersectionality, Gender, Migration, Solidarity practices

## Abstract

Nearly half of those who experience forced migration worldwide are women. These women often experience disempowerment and face distinct challenges in building their daily lives and participating in society. Factors such as gender, education, economic resources, age, and status intersect to shape their experiences. In this paper intersectionality and an occupational perspective on everyday life are used to analyse participation of women with forced migration experiences (WFMEs). A tactic-strategy continuum was applied to explore challenges and power dynamics in urban everyday lives among participants, focusing on how intersectionality shapes their engagement in new communities. Qualitative interviews were conducted in Sweden and Germany and analysed using reflexive thematic analysis. The findings are presented as four main themes highlighting experiences of intersectional power dynamics. Women employed tactics to move from uncertainty towards hope, which is illustrated through both disruptions and ongoing risks of precariousness, as well as the use of everyday occupations, such as bicycling and gardening. Social or strategic actions, such as building solidarity and individual negotiations, are presented as forms of tactics to manage uncertainty and hope. The findings indicate diverse use of power strategies and tactics which are used in the daily lives of WFMEs. Occupational therapists have the expertise to support WFMEs in rebuilding their daily lives within a new society. They can do so by using tactics such as bicycling and engaging in solidarity, which can help address power imbalances created by intersectional structural dynamics.

## Introduction

Since 2015, refugee applications in Europe have risen because of complex geopolitical and humanitarian crises. In 2024, about 120 million people were displaced due to war, conflict, or persecution. Nearly half of forcibly displaced people were women [1]. Since October 2023, the number of people seeking asylum in Europe has decreased due to stricter border controls and the EU Asylum and Migration Pact [2].

Women who have escaped conflicts and seek refuge in other countries face violence, discrimination, and gender-related health risks during their migration trajectory—before, during, and after leaving their country [3, 4]. In comparison to men, women seeking refugee status are at higher risk of post-traumatic stress disorder caused by gender-related violence, including sexual abuse, rape, and other forms of torture and serious gender violence [5–7]. “Gender-based violence (GBV) is violence committed against a person because of his or her sex or gender” [8]. Gender-based violence is caused by structural, political, economic, and social imbalances between men and women [9].

Shishehgar et al. [10] identified key factors affecting the resettlement and health conditions of women with acquired refugee status after migration, including access to healthcare services, language barriers, lack of housing, cultural factors, and gender roles. Although these factors affect people seeking refuge in general, they are particularly important for women seeking refuge because childcare responsibilities can limit access to language courses and education [6].

This study examines the everyday lives of women with forced migration experiences (WFME) and their social participation in a new society, focusing on two urban areas in two European countries with comparable welfare systems. Urban areas were chosen because people with forced migration experiences often move to cities for infrastructure and structural conditions that differ from those in rural areas. Urban areas offer better access to language courses, work, and public transport, but they also involve more bureaucracy and more competitive job and housing markets [11, 12].

An occupational lens helps explain how socio-structural conditions shape everyday life and how WFME can engage in meaningful occupations to create everyday life in a new society.

Since 2015, occupational therapists, researchers, and teachers from occupational therapy programs in Europe have responded to the European humanitarian crisis by establishing OT for Displaced Persons, a network that brings together occupational therapists working professionally or as volunteers with forcibly displaced individuals. Occupational therapy researchers and practitioners have addressed the health issues of people who have experienced forced migration by focusing on everyday life occupations [13–19].

Gupta and Sullivan [14] emphasised how forced migration changes WFMEs’ lives and identity in a new society. They identified various ways in which WFMEs pursue occupations that can either renew roles and identities or maintain habits and occupations related to everyday life in their country of origin. Occupations shifted in response to identity changes and the new socio-cultural environment [14].

This matters from an occupational perspective, as it shows how women’s everyday occupations are shaped by experiences of forced migration before, during, and after resettlement [4], as well as by socio-structural conditions in the new society. Through an occupational lens, the concepts of necropolitics [20] and slow violence [21] as presented by Mayblin et al. [22] illustrate how structural conditions and political power can challenge WFMEs’ efforts to rebuild everyday life.

### Understanding Everyday Life of WFMEs Through the Lens of Power

To strengthen the role of occupational therapy in research, practice, and education on forced migration, a nuanced understanding of everyday life experiences and opportunities for participation in the new society is essential. Building on this, this study draws on the “tactic–strategy continuum” introduced by Sereke and Drzewiecka [23], to examine how WFMEs shape their daily lives and to discuss power dynamics that influence their possibilities for participation in society. Sereke and Drzewiecka [23] conceptualise tactics and strategies “as a continuum [that] offers a useful heuristic for understanding how migrants deploy power and develop ‘techniques’ to overcome or sidestep barriers, negotiate strategies of state integration, and counter racist exclusions, calculating what will work and how” [23]. In this study, such “techniques” are understood as the everyday decisions, actions, and occupations that WFMEs use to navigate and balance within this continuum. The “tactic–strategy continuum” is grounded in De Certeau’s philosophical concepts of tactics and strategies [24]. De Certeau employed these concepts to demonstrate how power shapes everyday life. Strategies are understood as frameworks of actions used by organisations, institutions, or networks. Actors in powerful positions use strategies to organise and influence the conditions of everyday life for those within their sphere. For instance, migration offices in Sweden and Germany exert power by the use of certain strategies: they regulate access to housing, work, and health and social services for people with forced migration experiences [25, 26]. Tactics, in contrast, belong to people who build their everyday lives within these pre-established frameworks. People creatively use tactics to construct meaningful lives from less powerful positions as “the place of a tactic belongs to the other” [24]. Tactics refer to how individuals navigate spaces, contexts, and institutions that are shaped by others with more power. WFMEs arriving in Europe enter environments where they often occupy less powerful positions, for example, due to language barriers or the limited recognition of qualifications obtained elsewhere. They draw on their own resources—such as community engagement and social networks—as creative and flexible opportunities that are essential for constructing everyday life and participating within environments created by others [19, 27]. We follow Sereke and Drzewiecka in conceptualising tactics and strategies as points along a continuum of action, rather than as a binary opposition [23].

### Intersectionality and Social Participation

An additional theoretical resource used in this study is intersectionality as understood by Mohanty [28]. This framework will be applied throughout the study to guide reflexivity and reveal inequalities produced by overlapping power dynamics related to gender, religion, age, disability, and ethnicity [29–32], which impact the everyday lives of women who have experienced forced migration. Mohanty applies intersectionality within a global context that considers colonialism, patriarchy, and capitalism when examining how power structures position individuals with intersecting factors, such as gender, class, and ethnicity. Power and forms of resistance are integral to her work, as she describes solidarity practices among women [28].

Besides intersectionality, this study gives emphasis to social participation. According to Niemi et al. [33], participation includes three dimensions for those with migration experiences: (1) regulatory frameworks that set conditions for participation in society; (2) social organisations and structures providing access to health care, the labour market, and other participation opportunities; and (3) community-organised groups where people with forced migration experiences can engage.

## Aim

The overall aim of this study is to explore power dynamics in the everyday lives of WFMEs in an urban context through the lens of intersectionality, emphasising their social participation in the new community.

## Methodology

Experiences of WFMEs, embedded in their everyday lives, are understood from a constructivist view and integrated in this study as knowledge and stories [34]. The first author actively participated in the process of knowledge development to effectively interpret data in various situational and contextual circumstances. In this sense, the social interactions and experiences of the participants were understood as interconnected with those of the researcher [35].

Semi-structured interviews (Germany and Sweden) were conducted between August 2021 and October 2025. Three ethical permits were needed to collect data in two countries. Germany granted the first ethical approval in July 2021. Sweden granted the second in May 2025, and the final Swedish permit to use both datasets was granted in March 2026. Immediately after conducting the first interviews, the analysis process started iteratively.

The participants (Table 1) were between 21 and 49 years old, had forced migration experiences, and lived in urban areas in their new countries. The interviews were conducted by the first and last author in German, English, or Swedish. Additionally, one interview was conducted in Dari and two in Farsi with the support of interpreters and trained cultural brokers. Due to the COVID-19 pandemic, most interviews in Germany were conducted online or under restrictions, including the wearing of masks and maintaining social distancing. The interviews that included interpreters were translated to German or English. Cultural brokers were given more time for translation of these interviews to ensure accurate translations.

**Table 1:** Participant information

Participant (name changed)	Age, self-reported health issues, & level of education	Number of years in the new country
Yasmin	In her 30 s, has a disability, low education	2 years
Amira	In her 40 s, married, two children, mental health issues, a former specialist physician, volunteer work in an NGO for refugee women	9 years
Lina	In her 20 s, mental health issues, high education (master’s degree)	2 years
Tahani	In her 20 s, living with her mother, mental health issues, no formal education	1,5 years
Naima	In her 40 s, married, three children,, volunteer work in NGO for refugee women, no formal education	5 years
Dina	In her 30 s, two children, married, mental health issues, had own business in her country of origin	2 years
Eleen	In her 30 s, mental health issues, formal education	2 years
Sofia	In her 30 s, living with her partner, mental health issues, education in health care	3 years
Fayeza	In her 40 s, four children, widow, no formal education	2 years

The recruitment process involved contacting a psychosocial health centre in Germany and three networks of non-governmental organisations in Germany and Sweden that focus on forced migration issues. From these contacts, additional participants were recruited through snowball sampling [36]. Participant data was partially provided in aggregate here to maintain confidentiality to the greatest degree possible. All interviews were guided by an interview protocol that provided orientation while encouraging dialogue in the interview situation. The interviews lasted between 45 and 90 min. Face-to-face interviews were conducted in a location chosen by the participants.

All interviews were transcribed verbatim in the original language to maintain context and meaning. Interviews conducted in Swedish, Farsi, and Dari were translated into English. The translator/cultural broker checked the translated version content for accuracy rather than translating it verbatim. The analysis was conducted using codes in English; however, specific quotes were kept in the original language during the analysis for as long as possible.

### Researcher’s Positionality

The first author is a migratised female doctoral student, born and living in Germany with roots in Pakistan. She has worked as an occupational therapist and as a volunteer with people with forced migration experiences in Pakistan and Germany.

The second author is a male researcher with experience of voluntary circular migration between Sweden, Japan, and the USA. He grew up in an academic and multilingual context. He has a background in psychology, public health, occupational therapy, and occupational science. His work foregrounds participation and inclusion among persons risking exclusion.

The third author is a male researcher who grew up in southern Germany in a rural working-class environment. His academic background is in Social Geography, History, and Cultural Anthropology. His academic work has mainly been in the Critical Geography Network on the dialectics of space and power conducting research on racism and exclusion in small towns in East Germany.

The fourth author is a male researcher who has experienced migration between Chile, Sweden, Hungary, Germany, and Spain. He has a background in sociology and applied ethics and has engaged in research focusing on human rights and research ethics.

The last author is a female researcher who grew up during the dictatorship in Chile (1973–1989). In the post-dictatorship period, she worked in the field of human rights and mental health as a clinical occupational therapist, encountering people who had experienced exile, among other human rights violations. Later, she moved voluntarily to Sweden, and her scholarly work focuses on the intersections of human rights, health, and migration from an occupational perspective.

### Data Analysis

To explore the everyday life activities of WFMEs, focusing on social participation through the lens of intersectionality, a pluralistic data analysis was employed [32, 37]. A pluralistic data analysis enabled a comprehensive examination of a data set, in this case, semi-structured qualitative interviews, from multiple angles [37]. Therefore, a reflexive thematic analysis [35], which included intersectionality [28, 30–32, 38], was used. More concretely, intersectionality has been used as a theoretical lens for critical reflexivity across all six steps of reflective thematic analysis [39]. The six steps—familiarisation, initial coding, searching for themes and patterns, reviewing themes, defining and naming themes, and reporting findings—were not understood as a recipe to follow, but as an iterative analytical process [40]. After transcribing the interview data and reading the transcribed data done by the translators, the first author became familiar with the material by reading the interviews one by one and summarising their content [39]. In addition, field notes [41, 42] and postscripts have been considered to provide contextual and situational information to enhance the interviews [34]. Key observations and reflections from these documents were coded similarly to the interview transcripts, enabling an integrated analytical process that strengthened the overall rigour of the analysis. To facilitate the coding process, the computer-based program Atlas.Ti was used.

In line with reflexive thematic analysis, the study’s deductive orientation [43] was grounded in intersectional theory and theories about everyday life as proposed by De Certeau [24], elaborated with focus on people with migration experiences by Sereke and Drzewiecka [23], and connected to meaningful occupations as discussed in the field of occupational therapy and occupational science [44–46]. This theoretical underpinning allowed for identifying power dynamics in the everyday lives of WFMEs by focusing on everyday occupations, opportunities for participation, and disruptions in everyday life. Therefore, the codes have been categorised into intersectional dimensions, first one by one, for each participant, and second across all participants.

The codes were foundational to searching for potential themes by identifying thematic patterns. These patterns and potential themes were discussed and reviewed among the co-authors.

The data analysis and interpretation followed an iterative process, involving several analytical rounds between the first and last authors between August 2021 and December 2025. Through this reflexive process, meaning-based interpretative themes were created [40]. Meaning-based interpretative themes referred to the creation of themes based on meanings that arose during the analysis process. These themes consider the underlying assumptions, contextual factors, and the researchers’ reflexivity.

The first author was responsible for conducting the initial thematic coding and organising the data, while the last author, together with the first author, facilitated the intersectional analysis and ensured alignment with theoretical frameworks. The analytical engagement of all co-authors ensured a rigorous examination of the data.

## Ethical Considerations

All participants received written and oral information about the study in English, German, or Swedish. For those requiring interpretation, information was provided with interpreter support in Dari and Farsi. In accordance with the Declaration of Helsinki [47] and the International Ethical Guidelines for Health-Related Research Involving Humans [48], ethical procedures included obtaining written and oral consent, with participants able to withdraw from the study at any stage. Participants signed consent forms, which were stored, following the storage guidelines of the author’s researcher institute, confidentially by the first author. To prevent harm and ensure safety and confidentiality, all personal data were handled securely to maintain anonymity [49]. The first and last authors had therapeutic experience with forced migration and remained attentive to potential triggers during or after the interviews. Accordingly, each participant received a follow-up phone call several days after the interview. Contact information for psychosocial networks and NGOs supporting women with experiences of forced migration was also made available.

## Results

This study aimed to explore power dynamics in the everyday lives of women with experiences of forced migration in an urban context through the lens of intersectionality, emphasising their social participation in the new community.

The results are presented in four themes: (a) moving between uncertainty and hope; (b) disrupted narratives: the risk of precariousness in everyday live; (c) embracing nature-based occupations linked to freedom, gender equality, and health; and (d) engaging in solidarity.

The first theme illustrated how participants moved between uncertainty and hope in their daily lives through tactical choices within a society that holds strategic power, leaving them in an uncertain and powerless position. This unpredictability made social participation and a sense of stability in everyday life more complex.

The second theme addressed experiences of disruption in participants’ life narratives, including situations of precariousness that limited their ability to balance on the tactic-strategy continuum.

The third theme explored occupations such as bicycling and gardening as new, liberating, and meaningful occupations that can be seen as tactics to regain greater strategic power. The fourth theme, engaging in solidarity towards other women in precarious situations, reflects a form of resistance to oppressive forms of governing in everyday life.

### Moving Between Uncertainty and Hope

The participants’ everyday lives were strongly shaped by their forced migration trajectories and their status in the new country. In addition, national elections, the COVID-19 pandemic, and a right-wing turn in several European societies affected their daily lives. The participants employed powerful metaphors to express uncertainty and liminality, often linked to waiting, doubting, and being prevented from establishing a sense of stability in everyday life due to structural conditions and policies.

Their accounts further illustrated how language, education, family responsibilities, religion, experiences of forced migration, and mental health conditions intersect to influence movements between uncertainty and hope in everyday life. For these participants, uncertainty and hope arose both from the traces of experiences in their countries of origin and from the challenges and negotiations they faced in the present. Their everyday experiences can be understood as an ongoing negotiation, shaped primarily by migration regulations, the precarity of social networks, and the complexity of adapting to new societal norms.

It is my goal to stay in Germany, but I had to wait for a residence permit and to get access to a German language course for more than three years. When I obtained a temporary residence permit and began learning the language, I became ill for one or two years before resuming my language studies. Since I left (name of the country of origin) and came to Germany, I have had severedepression. No place to live, no work, no language.(Amira)

This example showed how interconnected factors like residence uncertainty, mental health issues, and limited language training opportunities can make daily life unbearable, if not impossible to manage. The findings highlighted the precarious and uncertain conditions faced by WFMEs. Without residence permits, participants described how this status concretely impacted and restricted their everyday lives. Reflecting this sense of uncertainty, Lina described a feeling of being “lost in the woods”, which depicts fear, liminality, and ambiguity. During this uncertain period, Lina considered forced displacement as “the hardest situation a human being experiences”, she felt torn between countries, “not there and not here at the same time – like a ghost”. Applying Sereke and Drzewiecka’s [23] understanding of power negotiated in everyday life, this uncertainty can be viewed as a strategy, where structural barriers prevent further participation. In contrast to disempowerment, Lina’s experience offered another perspective. She found room and work opportunities through her social network, enabling her to enjoy religious freedom and to deal with emotional challenges. In Lina’s everyday life, her social networks consisted mainly of the church and an NGO offering language courses, legal support, and consultation for people with experiences of forced migration. Within a tactic-strategy continuum as understood by Sereke and Drzewiecka [23], both the church and the NGO can be interpreted as organisations with greater strategic power that nevertheless serve Lina’s purposes. Her opportunity to build a network through these organisations can be understood as a tactic to improve the conditions for constructing her everyday life.

Tahani experienced structural limitations and discrimination based on her ethnicity, religion, gender, status, and lacked a broader social network. Social participation in Tahani’s everyday life was restricted because of these intersectional constraints:

I don’t get help when I ask people something—they just ignore me and don’t give information about where I should go. I’m unsure where to register my name for schools or who can provide me with information. And about buses—how do we take tickets? How can I go shopping at places like name of a grocery here? Without thisrefugeecentre, I don’t know of any other place to shop. (Tahani)

Tahani’s negative experiences can be interpreted as the consequence of having very limited everyday opportunities, which constrained her ability to employ tactics for navigating the new society. In contrast, uncertainty in the participants’ lives became less threatening, and hope emerged as they developed greater knowledge of the local language and learned to navigate new societal systems as forms of tactics. Acquiring language skills and information helped to clarify situations and reduce uncertainty. For Yasmin, “Information is more than money”. She sought help from an NGO and the church, where she received guidance about health, work, and Sweden’s social systems. Naima reflected that, in 2016, people from her country of origin did not have access to language courses. However, whenever she had the opportunity to learn new words and numbers used in Germany, she wrote them on the wall to memorise as many as possible. She also raised her hand, saying “I want, I want” when a person came to the refugee centre and asked, “Who wants a job?” The way Naima used every opportunity that she had previously been denied in other life contexts, such as work, can be understood as a tactic that enabled her to shift from uncertainty to hope. Moving between uncertainty and hope offers a way of understanding participants’ actions within a tactic-strategy continuum that is profoundly shaped by intersectional power dynamics.

### Disrupted Narratives: The Risk of Precariousness in Everyday Lives

Building an everyday life in a new society posed significant challenges for participants along their migration trajectory. These changes disrupted their established life narratives.

As previously discussed, participants navigated uncertainty and hope. Many felt powerless or lacked control, creating a sense of precariousness and making it difficult to establish meaningful daily lives in the new society. Disrupted narratives resulting from challenges faced related to work, education, and other meaningful occupations identified by participants were important findings for understanding how power dynamics in the new society shaped participants’ daily experiences.

Amira, a former specialist physician, emphasised that her career was central to her identity. She said, “I need to wear my white coat”. In Germany, she could not practice medicine and had to start over. She described it as being “like an illiterate”. She faced work restrictions due to her residence permit, which affected her mental health. She was allowed to work as a nursing assistant and described feeling like “a small human” compared to her training. Amira noted that a high level of education can make it harder to enter the labour market at the right level. Bureaucracy and professional validation processes took years. These processes operate as strategic mechanisms that shaped the possibilities for highly educated women to work in their professional fields.

Similarly, Sofia’s experience demonstrates the precariousness that WFMEs face due to restrictive Swedish work regulations. She experienced a lack of practical support and uncertainty surrounding her requalification as a midwife, which hindered her ability to reclaim her professional role. This uncertainty limits participants’ opportunities to continue working in their field or a related field. Together, these challenges highlight the complex journey that highly educated migrant women undertake to rebuild their careers and achieve recognition in a new society.

Eleen expressed a desire to participate in German society to repay for the support she has received. Therefore, she remained open to careers such as becoming a hairdresser or to work in the field of social care. However, her ambitions are challenged by the complex, lengthy process that people with experiences of forced migration face to regain professional and social standing. This includes completing language and integration courses and securing residence and work permits, which are closely interdependent. Additionally, she has to obtain language and education certificates to access the labour market. Fayeza, meanwhile, expressed a wish to “be a little useful and do something”, highlighting her awareness of her limited power to contribute within the system.

Some participants also experienced precariousness due to the intersection of gender roles, migration experience, family structure, and economic dependence. Dina and Sofia, for example, rely on partners and family for financial support and social interactions. Compared with her life in the country of origin, Dina felt unable to achieve social or economic independence because she was fully responsible for her children, lacked personal time, and could not build a social network outside her family. In her marriage, she felt more powerless since her husband was responsible for finances and major decisions.

Sofia’s relationship with a Swedish physician created a different form of precariousness. She relied on her partner for housing and finances and was unable to work as a midwife, describing feelings like a “small child”. This situation emphasises the power imbalance and disruption caused by such dependence.

Disruption and precariousness in participants’ everyday lives were also evident at the refugee centre. This institution holds strategic power and serves as a foundation for residents who have experienced forced migration as they rebuild their daily lives.

There is nothing in the camp, especially for women. Men can play. They were playing in (name of the refugee centre), but in the family camp, no one sees anyone outside their rooms. Everyone stays in their room with their children, using mobile phones, so they don’t come outside. Everyone is frustrated. No one even wants to talk. The workers are rude and don't like talking politely to anyone. (Tahani)

Tahani also described violence and discrimination involving other refugee men and German people living near the refugee centre. She experienced discriminatory treatment in both contexts.

Her suggestion for other refugee women who come to Germany was:

When a woman comes from another country and wears different clothes, I want to help her feel comfortable going out. Whatever she wears, she should feel confident and consider her clothing normal. I want people to feel more comfortable, and I aim to show them kindness.

By wearing a traditional Pakistani dress, Tahani’s mother experienced discrimination. Tahani’s suggestion addressed this issue and stressed the importance of empowering refugee women in Germany. Using dress as a tactic helps preserve identity and supports meaningful action.

### Embracing Nature-Based Occupations Linked to Freedom, GenderEquality, and Health

Bicycling and gardening stood out as everyday occupations with multiple meanings in the lives of refugee women. First, bicycling carried broad symbolic meaning associated with freedom, gender equality, and psychosocial well-being. Participants described diverse experiences associated with occupations of bicycling. For some, bicycling was important for their physical and mental health; for others, independence from previous gender restrictions and the experience of freedom were particularly salient. From an intersectional perspective, bicycling represents a taken-for-granted activity both in Germany and Sweden, an occupation that interconnects gender equality, health benefits, and is accessibly economically. It is a physical activity that can be enjoyed outdoors throughout the year and was perceived by participants as having a positive impact on both physical and mental well-being. Riding a bicycle can also be understood as a tactic that enables exploration of the environment, nature, neighbourhoods, and cities.

Tahani aspired to learn how to bicycle, an occupation that would allow her to explore the city—a freedom she was denied in her country of origin due to gender restrictions. Naima, who learned to bicycle in Germany, highlighted how this occupation empowers her and expressed a desire for other women from her country of origin to have access to the same opportunity. Her narrative illustrates how bicycling serves as a tactic to navigate independently in new neighbourhoods and to address the unequal power dynamics she experienced in her country of origin, where Afghan women were restricted from bicycling. Eleen, meanwhile, emphasised the positive impact of bicycling on her health and well-being. “I have tobicycle every day because of bad thoughts. I have nightmares” (Eleen).

Similarly, Sofia experienced bicycling for the first time in Sweden, having not done so in her country of origin. She, too, valued bicycling in her new context, appreciating both the freedom of movement it offers and its low cost, which was especially important when lacking income. Building on these experiences, the findings revealed that low-cost activities, such as bicycling, were vital for individuals with limited resources. Alongside bicycling, socialising in parks and participating in free activities offered by NGOs helped these participants to have meaningful encounters, build routines, and start rebuilding a nurturing social network. Besides bicycling, some participants engaged in gardening, which supported relationship-building within their social networks and promoted various aspects of health.

Dina and her family rented an allotment garden where they enjoy spending time together on weekends. For them, the garden symbolised a peaceful place of their own, where they could rest, tend to the plants, and be together as a family. Amira looked for an available green space in her neighbourhood where she could plant something for herself, creating a link between her present life and her past. Amira said, “I took a little bit of green space in my neighbourhood to plant a vegetable that is not available in Germany. It comes from(name of the country of origin)”. Amira used green space as a tactic to plant something that connected her to her cultural roots in the new neighbourhood. The green space that Amira cultivated, and Dina’s rented allotment garden, can be interpreted as tactics that shift the power dynamics in their neighbourhoods, as the participants described these places as peaceful and as their own. The findings illustrate how the participants’ occupations enable them to navigate their neighbourhoods and cities independently of their legal status, gender, or socio-economic circumstances. Furthermore, the freedom and mental well-being gained through these occupations can be understood as both physically and symbolically “gaining territory” in the new country, functioning as a tactic that counteracts structural barriers.

### Engaging in Solidarity

Empowering fellow WFMEs through non-governmental organisations and promoting a non-discriminatory perspective on women had a significant impact on everyday lives of several participants. Solidarity among WFMEs emerged as a salient dimension of their engagements and as a tactic embraced in daily life. Yasmin, for example, a woman with experiences of forced migration and a disability, participated in an organisation that supported refugees with disabilities and provided a space to connect, listen to one another, and take part in various activities together. Practical support from fellow members helped Yasmin to understand regulations for refugees with disabilities in Sweden and to find suitable housing. She observed that Swedish society respects and supports women with disabilities, which she appreciates.

Similarly, solidarity was central in the experiences of Naima and Amira, who were engaged as volunteers in an NGO that supported refugee women in Germany. Their involvement demonstrates how refugee women in different contexts support one another and address 15 shared challenges within the dominant system, using these actions as a form of resistance. This solidarity among women is further emphasised by Fayeza, who linked such engagement to the strengthening of their power positions through education and improvements in health.

*I’d focus on associations to bring women together, to help them learn the language and find work. These things change lives; they give people focus and peace of mind. I’d help illiterate and sick women.*‬‬‬‬‬‬‬‬

Naima enjoyed explaining her volunteer work, and shared what motivated her to support WFMEs, primarily from her country of origin.

*No matter if I get money or not, I want to help the women.*

Her experience highlights the diverse personal and professional outcomes of volunteer work. Through her volunteering, Naima became a more independent woman and securing a stronger position within the hegemonic social structures. However, this has not translated into economic stability. From her narrative, it became clear that women in her country of origin experience power imbalances due to gender-based restrictions [50]. Naima emphasised this when expressing her wish for other women from her country to experience a smoother transition and to find peace in their new country. Her volunteer work can be understood as a strategy to address structural barriers in the new society, including limited access to information, bureaucratic obstacles, and discrimination experienced both by her peers and herself, by guiding on how the system and society in Germany function. Naima’s engagement in volunteer work also aimed to address structural barriers imposed in her country of origin, where the issue of gender equality is characterised by restrictions in access to work and education, mobility, and experiences of forced marriage and other forms of structural violence [50]. Naima repeatedly emphasised the significance of freedom and gender equality, which she experienced firsthand upon arriving in Europe. She chose to move beyond the role of “housewife” as her primary identity and sought to support other WFMEs in accessing the same freedoms she had gained.

When I came to Greece, I shined and said to my husband: ‘I am in Europe, I am not just a housewife anymore.’

Gender equality was an important issue in Naima’s life but also for other participants. Equality was a goal not only for their own lives but also for the lives of other WFMEs, to prevent discrimination, persecution, and other forms of gender-based violence. Therefore,

also Amira actively offered counselling on topics such as breast cancer and contraception to WFMEs, helping them navigate the German healthcare system and understand the importance of health. This different content of counselling services underscores the varied effects of solidarity among WFMEs.

The intersection of gender, education, migration, health, and psychosocial well-being became evident in participants’ accounts of solidarity practices as volunteers, friends, and role models—for example, informing other WFMEs about the benefits of bicycling, supporting them in learning the German language, and engaging in shared activities such as gardening.

Drawing on the engagements of Naima and Amira with women-focused NGOs, together with those of their peers, we argue that WFMEs mobilise solidarity practices as tactics, alongside women who share similar experiences and commonalities, to counteract the lack of initiatives that promote and enable a good life for WFMEs.

Four themes emerged from the findings, highlighting intersectional power dynamics that affect WFMEs in urban areas. The use of tactics to move from uncertainty to hope was visible throughout the findings, as well as using tactics to deal with disruptions and the risk of precariousness. Occupations such as bicycling, gardening, solidarity, and individual negotiations stood out as ways to handle uncertainty and foster hope.

## Discussion

This study explored power dynamics in the everyday lives of WFMEs in an urban context through the lens of intersectionality, emphasising their social participation in the new community.

The findings demonstrated how power dynamics can shift in the participant’s everyday life due to structural inequalities that hinder social participation. Further, the findings reveal the use of tactics and solidarity practices to counteract and negotiate possibilities to construct everyday life under uncertain circumstances.

In the discussion, we connect these findings to Sereke and Drzewiecka’s [23] tactic-strategy continuum, which is rooted in De Certeau’s framework of tactics and strategies [24], along with occupations, such as bicycling and gardening, and intersectionality to illustrate the impact of power dynamics on daily life and participation. We explore how solidarity and other tactics manifest in the daily experiences of women with a history of forced migration.

### Intersectional Lens on Power Dynamics in the Everyday Life of WFMEs

Through an intersectional lens [51–53], it was possible to reflect on the participants’ everyday life experiences, which included disrupted narratives, a state of precariousness, and disempowering changes. Intersections of disability, gender, ethnicity, age, education, and socioeconomic status linked to power dynamics in everyday life caused by structural inequalities and institutional othering could be explored [54]. For example, work and education were key markers in the participants’ everyday lives in which the degree of education impacted strategic options. Yet, additional resources, e.g., voluntary work, were often overlooked. The intersection of gender, status, and education likely excluded WFMEs from full participation in work or society and disrupted their career paths. As a result, their engagements did not match their resources or goals.

Further, the participants’ everyday experiences revealed disempowerment in work-related occupations, within partner relationships, and when describing their dependence on institutions, such as living in a refugee centre. The term “small child” was used by participants as an expression of infantilisation created by limited and oppressed living conditions that impacted the possibility of constructing everyday life in the new society. This metaphor and the experience of disempowerment were in line with gender equality–based violence [5, 55] and gendered societal expectations about WFMEs’ role in the new society, including being in a financially dependent position, doing domestic care, or being responsible for childcare [56].

Experiences of disempowerment can be discussed further as consequences of slow violence [21], which is part of structural, institutional violence [57]. In this context, slow violence refers to the bureaucratic delays and sluggish process of obtaining a residence permit that would grant access to language courses and other benefits, helping WFMEs to feel a sense of progress, reducing uncertainty, and the fear of deportation. The WFMEs in this study had many ideas about constructing a meaningful life and about participating in society. However, due to slow violence [22] and structural inequalities that lacked consideration of intersectional issues, WFMEs experienced a sense of uncertain waiting caused by necropolitical structures [20, 22]. Necropolitics describes policies and structures that create unequal conditions, based on the idea that some lives are less valuable than others [20, 22]. What is less visible in this study, but has been highlighted in other work by Scheer et al. [27], is the unequal treatment of WFMEs from Ukraine compared with those from non-European countries. WFMEs from Ukraine have a faster track to apply for asylum and therefore more often could access health and social systems. Among WFMEs in Sweden and Germany, participants experienced that different “classes” of migrants exist, shaped by intersecting dimensions of ethnicity, religion, and gender [27].

### Occupations and Hope as Means to Dealing with Power Dynamics

Viewing power dynamics through an intersectional lens in relation to the tactic-strategy continuum perspective can be valuable [23]. On a tactic-strategy continuum—where “tactics” refer to everyday responses and “strategies” to long-term plans—disempowerment shapes how people participate in work and social systems.

Bicycling and gardening were identified as meaningful for the participants. Bicycling represents more than just a physical cost-free activity or a means to explore the city. It represents a part of women’s rights movement in which bicycling has played a significant role since the late nineteenth century [58]. Bicycling can be seen as a “tool for liberty” [58], fostering women's emancipation from breaking free from patriarchal social norms. In relation to intersectionality, it can serve as a tool that addresses liberation (gendered norms and socio-economics of transportation) while also contributing to health.

Bicycling has long held an important place within feminist movements and is increasingly addressed in projects that support refugee women in new societies [58]. One participant, for example, described her motivation to teach other refugee women to ride a bicycle as both an act of solidarity and a response to the freedom that bicycling symbolises. Mohammadi and Enderle further emphasise that bicycling not only promotes social participation in the new society but also contributes to reducing gender disparities [59, 60]. Though bicycling can be a “window of opportunity” [59], not all participants in this study have the opportunity to ride a bicycle due to intersecting factors such as socio-economic conditions or family responsibilities. From an occupational therapy perspective, bicycling can be a valued occupation for many people and has even been highlighted as a self-therapeutic option to enhance health and well-being, serving as a “feel-good factor” [61]. This aligns with Eleen’s quote about bicycling as a means to reduce mental health symptoms.

Some participants engaged in gardening as a meaningful occupation and enjoyed planting seeds or spending their free time with family and friends. In the context of forced migration, gardening as a meaningful occupation has been described by Ekstam et al. [62] as offering opportunities to rest and experience a safe place where people can actively engage in gardening, also together with others. Nature-based occupations such as gardening can be understood as forms of enacting change, both in individuals’ lives and in society [63]. How participants describe finding a piece of green space and cultivating it as their own powerfully illustrates how tactics enacted through meaningful occupations [44] can position WFMEs with greater power to act in society. Therefore, meaningful occupations in the context of forced migration are highly relevant, as they create possibilities for navigating and acting within unstable conditions.

The findings reveal how WFMEs in Sweden and Germany sought to construct an everyday life in a society not designed for them, moving along a tactic-strategy continuum. Confronted with uncertainty and hope related to migration regulations, complex societal norms, and limited access to information, the participants drew on social networks and non-governmental organisations to navigate the new society. Here, the importance of “hope” during the migration trajectory and in the process of starting a new life in the receiving society is described “as an emotion that enables (…) to navigate uncertainties” [64 p. 764]. Hope can also be understood as an act of solidarity that brings people in together to create lives worth living amid suffering through new and responsive social practices [65]. “Hope” can be a key to considering alternative pathways when uncertainty about the future exists [64]. Mondaca [66] emphasises the importance of critically reflecting on the roles of occupational science and occupational therapy in *doing hope*, through the creation of practices that foster resistance to oppressive systems. Collaborative actions and open research dialogues with persons experiencing marginalisation or in vulnerable positions allow reflection on how “hope” can lead to dignified and fulfilling lives for those living under uncertain and disempowering conditions [66]. The uncertainty and precarity that characterises hope is influenced by European policymakers and public discourses against migration, which affect refugees at an individual level [67]. Hope is, therefore, an important dimension to consider when discussing tactics within the tactic-strategy continuum. Also, the success of acting in the tactic-strategy continuum seems to depend on intersecting factors that provide flexibility for WFMEs’ everyday decisions.

### Fostering Social Participation Through Solidarity Practices

According to the findings, solidarity practices supported other women in relation to their health, family issues, disabilities, and language education, helping them to establish a more independent daily life in the new society. Non-governmental organisations that provide safe spaces to meet, learn the language, and engage in shared activities offer important opportunities for such solidarity practices, as illustrated through initiatives such as bicycling and healthcare education for women with experiences of forced migration. This is interesting to reflect on community-organised groups as one dimension for social participation [33]. Solidarity practices in connection with NGOs, as well as among WFME groups themselves, are an important resource for creating everyday life under conditions of constraint and unequal power dynamics. It can therefore be important to consider safe spaces and opportunities for dialogue and exchange where WFMEs can engage in such solidarity practices [27].

In connection with NGOs, WFMEs can act more strategically. Moreover, solidarity practices were evident when participants received information about services and structures in the new society from other refugees. Within feminist movements, the practice of cross-cultural solidarity has been examined [28]. Mohanty [28] illustrates how women experiencing marginalisation in different countries use solidarity practices as a form of resistance, for example, through cooking together in a factory to emphasise their roles as working women and as housewives. Solidarity practices among marginalised women can also lead to larger movements that aim to achieve better work conditions. Solidarity is a counteract to neoliberalism and capitalism, which have created silos of marginality. Moreover, solidarity practices are visible across different layers, illuminating multiple intersections when focusing on working women. Gender, ethnicity, socio-economic status, family responsibilities, and different attitudes towards work and gender roles are evident in her research on solidarity practices [28]. Solidarity practices identified in the findings include providing health support, sharing skills and knowledge, and helping in navigating the new country’s system and society. For WFMEs with disabilities, barriers to the health and social care system become especially challenging [19, 68]. The findings clarified how disability and gender intersect, underscoring the need to address these intersecting factors in health and social policy and highlighting solidarity practices as essential for providing tailored support.

Solidarity practices in everyday life can be seen as actions along a continuum between tactics and strategies [23, 24], as forms of resistance [28], a collective occupation [69], and enabling hope [66] to overcome intersectional inequalities caused by structural violence.

## Methodological Considerations

This study employed a qualitative research design, utilising reflexive thematic analysis [35, 39, 40, 43, 70–72] and intersectionality as a critical reflexivity tool [28, 30–32]. In total, nine women with experiences of forced migration were interviewed in urban areas in Sweden and Germany. The interviews were partly conducted online due to COVID-19 restrictions, which can be seen as a limitation. Video calling was used to help capture the interview situation. For example, one participant used her mobile phone during the interview to show her apartment. Another possible limitation is the timing of data collection. However, the longer timeframe can be seen as a strength, as policy changes occurred over the years, affecting participants’ everyday conditions. Moreover, three interviews were conducted with the support of translators. The interviewers were experienced in using interpreters and aware of possible pitfalls. Therefore, the interviewers made sure to speak directly to the participant instead of the interpreter, observing mimicry and gestures and summarising the content to ensure mutual understanding [73, 74]. The inclusion criteria for the interpreters were that they had the same sex and ethnic background as the participant and that they had no personal relation to the participant. However, because the interviews were interpreted in phrases that reflected the content, the questions sometimes needed more detailed explanation to ensure the participant understood them correctly [75]. Regular analysis meetings with the research team, along with other analytical meetings between the first and last author, were conducted to capture the richness of the material and ensure trustworthiness [76]. Moreover, the analysis meetings were guided by critical reflexivity, which is essential in reflexive thematic analysis. Through an intersectional lens, we acknowledged our diverse professional and cultural backgrounds and how this influenced our interpretations. An advantage of this study is that interviews were conducted over a long period of time, allowing us to capture contextual factors such as election results, changes in migration policies, and the public discourse on migration, key aspects affecting the participants’ everyday lives.

Participant recruitment was based on information power [77]. During the study, the research team collectively decided to include refugee women from Ukraine in the data collection after the onset of war. The benefit of including women from Ukraine was gaining additional insights from participants who had more recently arrived in Sweden or Germany.

The quality of this study was established by applying Tracey’s [76] eight criteria for qualitative research: worthy topic, rich rigour, sincerity, credibility, resonance, significance, ethics, and meaningful coherence. The investigation of power dynamics and everyday tactics among women with experiences of forced migration is significant as it addresses intersectional inequalities, social participation, and access to meaningful occupations. Rigour was ensured throughout the entire research process. For example, using flexible semi-structured interviews, follow-up questions were employed, and summarised responses were used to accurately capture participants’ intended meanings. Sincerity was addressed through the authors’ reflexivity and positionality, which informed the authors’ understanding of power imbalances during recruitment, data collection, and analysis. Credibility was enhanced by employing reflexive thematic analysis, integrating intersectionality, and considering participants’ contextual factors. The inclusion of participants’ individual accounts in the results fosters resonance with readers at theoretical, practical, and emotional levels. Meaningful coherence was achieved by consistently applying an intersectional lens and theories of everyday life to analyse the results in relation to the research question.

## Implications

Because migration policies can limit everyday occupations and create power imbalances and intersectional inequalities, occupational therapy’s role in advocacy and empowerment may be important. Occupational therapy has the potential and resources in research, theory, and practice to act at multiple levels to dismantle existing inequalities for WFMEs that affect social participation, aligning with an intersectional understanding of overlapping structural barriers. Occupational therapists can benefit from incorporating an intersectional lens to understand overlapping issues that make meaningful occupations inaccessible. Those who meet WFMEs in clinical practice can benefit by understanding everyday life as tactics and strategies in motion, shaped by power dynamics, which could enable WFMEs to use their resources, knowledge, and ideas to strengthen strategies for successful social participation. More importantly, this understanding of everyday life requires occupational therapists to become allies or advocates for persons experiencing structural oppression rather than reproducing power imbalances in practice.

## Conclusion

This study explored power dynamics in the everyday lives of WFMEs in an urban context through the lens of intersectionality, emphasising their social participation in the new community. The results highlighted solidarity practices and nature-based occupations, such as bicycling and gardening, as important for constructing everyday life from experiences of an uncertain and position of less power in society. The participants described experiences of structural inequality, uncertainty, and othering caused by socio-structural power relations that hinder social participation. Within a tactic-strategy continuum, the participants used occupations as tactics to actively engage with the new society. In the everyday lives and social participation of WFMEs, occupational therapists could play a key role in recognising these structural mechanisms hindering participation, by taking intersectional power dynamics into account and by recognising occupations and solidarity practices as tactics expressed in everyday life.

## Data Availability

The dataset underlying this article is not publicly available but can be shared on reasonable request from the corresponding author.
